# Extraction and Separation of Active Ingredients in *Schisandra chinensis* (Turcz.) Baill and the Study of their Antifungal Effects

**DOI:** 10.1371/journal.pone.0154731

**Published:** 2016-05-06

**Authors:** Haijing Yi, Yan Chen, Jun Liu, Jie Zhang, Wei Guo, Weilie Xiao, Yuncong Yao

**Affiliations:** 1 Plant Science and Technology College, Beijing University of Agriculture, Beijing, 102206, China; 2 Beijing Key Laboratory of New Technology in Agriculture Application, Beijing University of Agriculture, 102206, Beijing, China; 3 Institute of Microbiology, Chinese Academy of Sciences, 100101, Beijing, China; 4 State Key Laboratory of Phytochemistry and Plant Resources in West China, Kunming Institute of Botany, Chinese Academy of Sciences, Kunming, 650201, China; The University of Wisconsin - Madison, UNITED STATES

## Abstract

*Schisandra chinensis* extracts (SEs) have traditionally been used as an oriental medicine for the treatment of various human diseases, however, their further application in the biocontrol of plant disease remains poorly understood. This study was conducted to develop eco-friendly botanical pesticides from extracts of *S*. *chinensis* and assess whether they could play a key role in plant disease defense. Concentrated active fractions (SE-I, SE-II, and SE-III) were obtained from *S*. *chinensis* via specific extraction and separation. Then, lignan-like substances, such as Schisanhenol B, were detected via High-Performance Liquid Chromatography-ElectroSpray Ionization-Mass Spectrometry (HPLC-ESI-MS) analyses of the active fractions. Moreover, the results from biological tests on colony growth inhibition and spore germination indicated that SE-I, SE-II, and SE-III could inhibit hyphal growth and spore generation of three important plant pathogenic fungi (*Monilinia fructicola*, *Fusarium oxysporum*, and *Botryosphaeria dothidea*). The study of the mechanisms of resistant fungi revealed that the oxidation resistance system, including reactive oxygen species (ROS), malondialdehyde (MDA), catalase (CAT), and superoxide dismutase (SOD), was activated. The expression of genes related to defense, such as pathogenesis-related protein (PR4), α-farnesene synthase (AFS), polyphenol oxidase (PPO), and phenylalanine ammonia lyase (PAL) were shown to be up-regulated after treatment with SEs, which suggested an increase in apple immunity and that fruits were induced to effectively defend against the infection of pathogenic fungi (*B*. *dothidea*). This study revealed that SEs and their lignans represent promising resources for the development of safe, effective, and multi-targeted agents against pathogenic fungi.

## Introduction

Bioactive substances derived from plants play important roles in environmental improvement, food production, food security, and biodiversity conservation. Increasing attention is being paid to the development and application of botanical pesticides, such as botanical insecticides, fungicides, antiseptic agents, and herbicides. An important reason for this interest is that botanical pesticides are accepted as being more environmentally friendly than synthetic organic pesticides with high residues, which can cause environmental pollution, threats to human health, and other issues [1–5].

Over the years, research regarding the application of botanical pesticides has shown that such substances are advantageous because of their high selectivity, low toxicity to humans, and biodegradability. Today they are one of the most important potential sources for the creation of new pesticides [6–9]. Several studies have shown that plant extracts contain active antibacterial ingredients, many of which have been extracted, developed, and applied as botanical fungicides [10–13]. However, identifying which species in the voluminous plant kingdom contain effective concentrations of active substances, and which of the ingredients in the 400,000 known secondary metabolites derived from plants have insecticidal activity [14], has been and will continue to be a puzzle to be solved.

In the USA, there are currently more than 245 registered biopesticide-active ingredients used in hundreds of products. In Korea, 33 biopesticides are registered, of which 19 are fungicides, 13 are insecticides, and 1 is an herbicide [15]. Industrial versions of ingredients, such as azadirachtin, matrine, rotenone, nicotine, celangulin, veratramine, ethylicin, physcion, aconitine, and eucalyptol have become the backbone of the botanical pesticide industry [10]. These substances are principally applied for their insecticidal effects. They eliminate pests through mechanisms such as acute poisoning, shutdown of the digestive system, hunger inhibition, sterilization, growth inhibition, and by acting as an attractant or repellent for the pest species. Some of these mechanisms are also active in the antiseptic and antibacterial processes of the botanical pesticides. The study of the effectiveness of botanical fungicides against crop disease remains at an early stage, and hence only a few marketable botanical fungicides have been developed thus far. To develop commercially competitive botanical fungicides, additional basic research into active antimicrobial substances is urgently needed. Thus, the immediate problem to be addressed is how to extract active ingredients from plants, develop new botanical fungicides, and improve plant disease control.

Family Schisandraceae, in the sub-class Magnoliidae, are perennial deciduous vines[16]. Plants of this family have been commonly used as medicinal and ornamental landscape plants. The family, comprising approximately 60 species, includes two genera, *Schisandra* Michx and *Kadsura* Kaempf Ex Juss. They are distributed discontinuously in Southeast Asia and North America [17]. In China, they are the most abundant plant resources and *S*. *chinensis* (Turcz.) Baill is a common species in northeastern China. The fruits, seeds, shoots, and leaves of *S*. *chinensis* (Turcz.) Baill have been used in traditional Chinese medicine since ancient times and are valued for their various curative properties [18]. The use of *S*. *chinensis* as a high-grade herbal medicine was first recorded in the ancient pharmaceutical book, “The Divine Husbandman's Herbal Foundation Canon (Shén Nóng Běn Cǎo Jīng)” [19]. Recently, *S*. *chinensis* fruit extract (SE) and its ingredients have gained substantial attention. They may play a potentially important role in the treatment of cardiovascular diseases, such as hypertension and myocardial infarction [20, 21]. A water decoction of *S*. *chinensis* is believed to act as an astringent for the lungs and kidneys. It is effective in the treatment of diarrhea, arresting excessive sweating, calming the spirit by refreshing the heart and kidneys, generating body fluid, and reducing thirst [22, 23]. Pharmacological studies on animals have shown that *S*. *chinensis* increases immunity and affords a stress-protective effect against a broad spectrum of harmful factors, including heat shock, sunburn, hypothermia, frostbite, aseptic inflammation, irradiation, and heavy metal intoxication [24, 25]. Previous studies have also proven that *Schisandra* contains numerous special kinds of lignans and organic acids. These include Schizandrin, deoxyschizandrin, γ-Schizandrin, isoschizandrin, Schizandrol A and B, schisantherin A-E, citricacid, malicacid, and tartaricacid, substances that could play a key role in the treatment of fatty liver, hepatitis virus infection, and chemical-induced hepatitis [26, 27]. They also help to relieve stress and anxiety [28–30], and increase insulin sensitivity [31].

However, current research on the *Schisandra* genus has primarily focused on species differences, medicinal ingredients, and pharmacological effects [32–35]. Less research has focused on how *S*. *chinensis* can be applied in agriculture to inhibit plant pathogens or its potential for the development of botanical fungicides. In this study, a new method of extracting active substances from *S*. *chinensis* is proposed and the effects of these extracts on *Botryosphaeria dothidea*, *Monilinia fructicola*, and *Fusarium oxysporu*m are tested, as these are causal agents of three important diseases (apple ring rot, peach brown rot, and *Fusarium* wilt of strawberry, respectively) [36–38]. The tests included the detection of the effects of the extract on mycelial growth, spore germination, and disease infection. Moreover, active ingredients in the *S*. *chinensis* extracts that inhibit the three plant pathogens were determined, and the mechanism of inhibition employed by the pathogenic fungi was studied.

Therefore, this study is meaningful because it produced alternatives to chemical pesticides and thereby could reduce the negative impacts of chemical pesticide use. It highlights the potential for development of botanical pesticides from *S*. *chinensis* compounds.

## Materials and Methods

### Plant and microbes materials

The experimental material was annual branches and leaves of *S*. *chinensis* collected from Hong You *Schisandra chinensis* Farm (124.8°E; 40.8°N) (http://www.hywwz.cn; Kuandian County, Liaoning Province, China). Samples of *Botryosphaeria dothidea*, *Monilinia fructicola*, and *Fusarium oxysporum* were obtained from the Plant Pathology Laboratory of Beijing University of Agriculture. They were stored in a refrigerator at a constant temperature of 4°C. Apple fruits (*Malus domestica* ‘Fuji’/*Malus prunifolia* (Willd.) Borkh) were obtained from the Chang Ping District (116.23°E, 40.22°N), Beijing, China. Peach fruits (*Prunus persica*) were obtained from Qingzhou City (118.4° E, 36.4°N) in Shandong, China.

Ethics Statement:The study was carried out on private land called the Hong You *Schisandra chinensis* Farm (http://www.hywwz.cn). We confirm that the owner of the land, Guangming Lin, gave permission for his site to be sampled. No specific permissions were required for locations and field studies, as our study did not involve endangered or protected species.

### Experimental design

#### Extraction and separation of active substances from S. chinensis

To completely remove moisture, the annual branches and leaves of *S*. *chinensis* were dried at 28°C for 10 days. Ten kilograms of air-dried materials were weighed and crushed into powder with a grinder (Huangcheng HC-1500Y2, Yongkang, China; http://shop1396630696864.cn.china.cn/supply/). Next, the powdered materials was mixed with 70% acetone at a powder:solution ratio of 1:2 at room temperature in a 70-L stainless steel container (http://www.jmxwm.com/xwm/en/index.asp; Xinweiming Stainless Steel Products Factory, Jiangmen, China). After 24 h, the solution was transferred to another container and an additional 70% acetone was added. This procedure was repeated three times, after which the soaking solution was placed in a rotary evaporator to distill at 40°C and reduced pressure (Büchi Rotavapor R-220, Essen, Germany).

The remaining water solution contained all the active substances extracted from *S*. *chinensis*. To filter impurities, the water solution was allowed to stand overnight and then filtered using a circulating water vacuum pump (http://www.gyyuhua.com; Gongyi City Yuhua Instrument Co., Gongyi, China). The filtering process was repeated three times. The filtrate was then extracted three times with pure ethyl acetate with a filtrate:ethyl acetate ratio of 1:2. The organic layer was separated from the aqueous layer and was concentrated by evaporating the organic solvent *in vacuo* at 40°C in a rotary vacuum evaporator (Büchi Rotavapor R-124, Essen, Germany). This process produced the primary crude extract, which was further separated along a gradient according to polarity and extraction rate. The primary crude extract was then accurately calculated. The primary extract totaled 240 g and all samples were stored in air-tight brown bottles at 4°C in a refrigerator for the duration of the subsequent experiments.

A portion of the extract (100 g) was subjected to separation using silica gel (200–300 mesh) column chromatography (CC) and sequentially eluted with a series of chloroform-acetone-methanol mixtures (1:0:0, 10:1:0, 9:1:0, 2:1:0 1:1:0, 0:1:0, and 0:0:1) to obtains seven fractions (from SE-I to SE-VII) (Table 1). For set SE-I, for example, the chromatographic column was eluted with chloroform at 20 mL min^-1^ for 2 h, and all the eluent was concentrated in the vacuum distillation unit (http://www.gyyuhua.com; Gongyi City Yuhua Instrument Co., Gongyi, China).

**Table 1:** Method of separating active substances extracted from *S*. *chinensis*.

*S*. *chinensis* extract	Organic solvent ratio	Elution time (h)	Velocity of flow(mL min^-1^)
SE-I	Chloroform	2	20
SE-II	Chloroform:Acetone = 10:1	2	20
SE-III	Chloroform:Acetone = 9:1	2.5	20
SE-IV	Chloroform:Acetone = 2:1	1.5	20
SE-V	Chloroform:Acetone = 1:1	1	20
SE-VI	Acetone	0.8	20
SE-VII	Methanol	0.8	20

The elution time and velocity of flow to obtain all the factions are listed in Table 1. The seven fractions were concentrated *in vacuo* at 35°C in the vacuum rotary evaporator (Büchi Rotavapor R-124, Essen, Germany). The yield of the seven fractions from SE-Ito SE-VII was 5.1 g, 6.6 g, 8.2 g, 7.1 g, 10.2 g, 6.9 g, and 13.1 g, respectively.

#### Preparation of spore suspensions of the three fungi strains

The three test strains were inoculated on potato dextrose agar medium (PDA) and cultivated in a biochemical incubator (Fuma SPX-160B-2, Shanghai, China) at 28°C with alternating light and dark every 12 h for 4 d. Following this, sterile water was continually added to the medium to wash off and collect the conidia of the fungi. To remove fragments of mycelium and medium, the solution was filtered through gauze and condensed in a centrifuge (Sigma 3K-15, Osterode am Harz, Germany) at 1,000 r min^-1^ for 1 min. The concentration of spores was then determined under a microscope (Leica DM750, Wetzlar, Germany) and adjusted to 1 × 103 spores mL-1.

#### Analysis of the mycelial growth rate of the three pathogenic fungi

Fractions from SE-Ito SE-VII (1 g) were dissolved in 1 mL dimethyl sulfoxide (DMSO) and the mother liquor (1g mL^-1^) for each fraction was obtained. Sterile liquid PDA (3 mL) was placed in each well of the six-well culture plate and different volumes (1, 10, or 20 μL) of mother liquor of extracts were added. The final concentrations of the medium-containing extracts were 0.3, 3, and 6 mg mL^-1^, respectively. Control wells contained the same volumes of dimethyl sulfoxide (DMSO). All concentrations were run in triplicate. All solutions were allowed to solidify for 4 h and a 10-μL suspension of different pathogen spores (1× 10^3^ spores mL^-1^) was added to the center of each SE-containing medium. After 3 h, the water in the spore suspension had completely evaporated and all six wells in the culture plates were placed in an incubator (Fuma SPX-160B-2, Shanghai, China) at 28°C for 48 h. The colony diameter was measured three times by the crossing method, and inhibition rates were calculated for each pathogens and extracts using the following equation1:${\text{E}}_{1}\;=\;\frac{\text{CC}-\text{CT}}{\text{CC}}*100\text{\%}$

E_1_, The inhibition rate of mycelial growth

CC, Colony diameter of control group

CT, Colony diameter of treatment group

#### Observing mycelial morphology with scanning electron microscopy (SEM)

Scanning electron microscopy (SEM) was performed to determine morphological changes in the mycelia on the treated media. First, the growing mycelia (3mm × 5mm) on medium treated with SE-III were sampled with a double-sided knife, and samples for SEM were fixed in 2.5% glutaraldehyde stationary liquid for 12 h. Next, samples were irrigation three times with 0.1 mol L^-1^ phosphate buffer. The samples were dehydrated with a gradient concentration of alcohol systematically at 30%, 50%, 70%, 80%, 90%, 95%, and 100% with 15 min at each gradient. Anhydrous acetone was then applied three times for dehydration and each bout lasted 15 min. A critical point drying apparatus (EMS 850, Pennsylvania, USA) was used to dry the sample completely. The dried sample was then pasted onto the sample stage to be sputtered with gold (Hitachi ion sputter E-1010, Tokyo, Japan) and observed via SEM (Tescan VEGA TS 5136XM, Brno, Czech) under 2,500× magnification.

#### Analysis of the spore generation rate of the pathogenic fungi

The method for preparation of the mother liquor (1 g mL-1) of every fraction was the same as described above. Spore suspensions (0.5 mL) of the three fungi (*B*. *dothidea*, *M*. *fructicola*, and *F*. *oxysporu*m) were mixed with 0.1 mL of 1 g mL ^-1^ SE-I, SE-II, and SE-III, respectively, and the mixtures were then cultured on a concave slide to retain moisture at 28°C for 24–48 h. The same volume of DMSO was mixed with spore suspensions as a control. Each treatment was replicated three times. Spore germination was observed under a microscope (40×, Leica-DM750, Wetzlar, Germany) after 24 h and 48 h. Inhibition rate of spore germination was calculated by the following equation2:

${\text{E}}_{2}\;=\;\frac{\text{TC}-\text{NG}}{\text{TC}}*100\text{\%}$

E_2_, The inhibition rate of spore germination

TC, Total number of checked spores

NG, Number of germinated spores

The minimum number of spores examined was 200. Germination was defined as occurring when the germ tube of the conidia was longer than the short radius of the spore.

#### Analysis of the infection status of the fruit pericarp by pathogenic fungi

The extracts SE-I, SE-II, and SE-III in an amount of 5 μL (0.1 g mL^-1^) were sprayed evenly on the pericarp of apple and peach fruits in a 3-cm diameter circle. The control group was treated with a corresponding volume of DMSO and all sprayed fruits were placed in a biochemical incubator (Fuma SPX-160B-2, Shanghai, China) at 28°C. Each treatment was replicated a total of 12 times for each of the four processing periods (0 h, 24 h, 48 h, and 72 h). After the processing period elapsed, a 0.1-cm sample of the apple pericarp was taken from the sprayed area and all samples were frozen in-80°C liquid nitrogen for the next analysis of ROS, SOD, CAT, and MDA.

Pathogenic fungi was activated and cultivated for 4 d and 0.5-cm diameter colonies were transplanted by pasting them on the center of the sprayed area. This process was repeated for pathogenic fungi corresponding to extracts of SE-I, SE-II, and SE-III, as described above. The apples and peaches were wrapped with cling film to retain moisture and then placed in a biochemical incubator (Fuma SPX-160B-2, Shanghai, China) at 28°C for 96 h. Observations took place 3 d later. To test the protective effect of the SEs in preventing *M*. *fructicola* and *B*. *dothidea* from infecting peach and apple fruits, the average diameters of lesions were measured.

### Antioxidant analysis

#### Measurement of intracellular reactive oxygen species (ROS)

A Reactive Oxygen Species Assay Kit (Nanjing Jiancheng Bioengineering Institute, Nanjing, China) was used to measure the level of intracellular reactive oxygen species (ROS) following the manufacturer’s instructions. The probe 2',7'-dichlorofluorescin diacetate (DCFH-DA) is oxidized by ROS to 2′, 7′-dichlorofluorescein (DCF), which is highly fluorescent at 530 nm. Briefly, a homogenate of apple pericarp was mixed with 1 mol L^-1^ DCFH-DA (V/V = 19:1). The relative levels of fluorescence were quantified by a multi-detection microplate reader with 485 nm excitation and 535 nm emission (Thermo Scientific, Varioskan Flash).

#### Thiobarbituric acid (TBA) test for lipid peroxidation

A Malondialdehyde (MDA) Assay Kit (Nanjing Jiancheng Bioengineering Institute) was used to measure the level of lipid peroxidation following the manufacturer’s instructions. MDA, the product of lipid peroxide degradation, can condense with thiobarbituric acid (TBA) to form red substances that are highly fluorescent at 532 nm.

#### Measurement of Superoxide Dismutase (SOD)

A Superoxide Dismutase (SOD) Assay Kit (Nanjing Jiancheng Bioengineering Institute) was used to measure the level of intracellular ROS following the manufacturer’s instructions.

#### Measurement of Catalase (CAT)

A Measurement of Catalase (CAT) Assay Kit (Nanjing Jiancheng Bioengineering Institute) was applied to measure the level of intracellular ROS following the manufacturer’s instructions.

#### Total RNA extraction and real-time PCR analysis

Total RNA was extracted from the pericarp of apples treated by the SEs for 0 h, 24 h, 48 h, and 72 h using a RN42-EASYspin Plus Plant Total RNA Kit (Aidlab, Beijing, China) according to the manufacturer’s instructions and then treated with DNase I (TaKaRa, Tokyo, Japan) to remove the residual DNA. The total RNA was then subjected to a reverse transcription reaction to generate first-strand cDNA using oligo (dT) primers (TaKaRa, Tokyo, Japan).

Expression analysis of genes was conducted by real-time PCR with SYBR Green qPCR Mix (TaKaRa, Tokyo, Japan) and a Bio-Rad CFX96 Real-Time PCR System (BIO-RAD, Hercules, USA). Actin (GenBank accession number: DQ341382) was selected as a ribosomal reference gene. Sequences of genes *MdPR4*, *MdAFS*, *MdPPO*, and *MdPAL* special primers used in this study are listed in S1 Table. The PCR mixture (20 μL total volume) for the reaction contained 10 μL SYBR Green Mix, 2 μL cDNA (diluted 10 times), 1 μL of each primer (10 μM), and 6 μL RNase-free water. Samples were amplified for 40 cycles at 95°C for 15 s and at 59°C for 20 s, and the reactions were all run with three biological replicates and two technical replicates. The real-time PCR data were calculated using the 2^ (-ΔΔCt) analysis method with error bars representing the standard deviation (SD).

#### HPLC-ESI (±)-MS2 analysis and identification of lignans

High-Performance Liquid Chromatography-ElectroSpray Ionization-Mass Spectrometry (HPLC-ESI-MS) analyses of the lignans were performed using an Agilent-1100 HPLC system equipped with a UV detector coupled to a LC-MSD Trap VL ion-trap mass spectrometer with an ESI source (Agilent Technologies, Palo Alto, CA, USA). The HPLC separation conditions were identical to those used for HPLC-DAD analysis. Negative-ion (PI) mode was used for the MS analysis of lignans.

ESI was performed using the following conditions: capillary voltage, 5.0 kV; nebulization pressure, 241.3 kPa; gas (N^2^) temperature, 350°C; and flow rate, 8.0 L min^-1^. The capillary offset and exit voltage were 68.4 V and 100.6 V, respectively. The skim 1 voltage was 29.5 V, the skim 2 voltage was 6.0 V, and the MS and MS^2^ spectra were recorded over an m/z range of 150 to 2000 NI. The MS^2^ scan of the target ions used normalized collision energy of 20%–45%. The experimental standards of Schizandrol A, Schizandrol B, Schizandrin B, Schizandrin A, and isoschizandrin were purchased from Sigma-Aldrich (Steinheim, Germany); data obtained for experimental standards and information regarding the identified compounds were combined to identify the lignans.

### Data Analysis

Three replicates were performed for each experiment. Data are presented as the mean ± standard deviation (SD). Experimental data processing and analysis were completed using SPSS 20.0 (SPSS Inc., Chicago IL, USA). Analysis of variance of all values was used to assess differences in the means among samples (P < 0.05). The virulence regression equation was also calculated using SPSS 20.0. Inhibition ratio was converted to the probability value (y) and concentration of the SEs was converted to logarithm (x). A virulence regression equation is then calculated as y = a + bx, where “a” is the intercept and “b” is slope. According to the equation, the EC_50_ value was calculated. Graphs were prepared in OriginPro 8.0 software (Origin Lab Corporation, Northampton, MA, USA).

## Results

### The inhibiting effect of SE-I to SE-VII on the mycelial growth of three pathogenic fungi

In the study, according to gradient elution, seven fractions were obtained from crude *S*. *chinensis* extract and were named SE-I, SE-II, SE-III, SE-IV, SE-V, SE-VI, and SE-VII (SE series, SEs) according to polarity sequence.

In order to investigate the inhibiting effect of SE-I to SE-VII on the mycelial growth of *M*. *fructicola*, *F*. *oxysporum*, and *B*. *dothidea*, antifungal tests with different concentrations (0.3 mg L^-1^, 3 mg L^-1^, and 6 mg L^-1^) of the SE series using the plate cultivation method were conducted. The results indicated that SE-I to SE-III had inhibitive effects against *M*. *fructicola*, *F*. *oxysporum*, and *B*. *dothidea*, but their activities varied among the different pathogenic fungi, with the inhibitive ability of SE-III being best of the three candidate fractions based on their EC_50_ values. However, SE-IV to SE-VII had no critical impact on the growth of pathogenic fungi (Table 2).

**Table 2:** Percentage of inhibitive effect for seven SEs on the mycelial growth of three pathogenic fungi.

*Schisandra* extract	Strains	Concentration			Toxicity regression equation	r	EC_50_(mg/mL)	EC_50_ ($\bar{x}$ ± SD /(mg/mL)
		0.3mg mL^-1^ 3 mg mL^-1^ 6mg mL^-1^						
	*M*. *fructicola*	23.21%	47.27%	50.94%	y = 4.59 + 0.60x	0.99	4.96	
**SE-I**	*F*. *oxysporum*	26.90%	44.44%	51.39	y = 4.65 + 0.48x	1.00	5.54	4.67 ± 1.05 (**P = 0.01**)
	*B*. *dothidea*	29.25%	39.63%	63.55%	y = 4.70 + 0.59x	0.87	3.51	
	*M*. *fructicola*	25.00%	41.82%	58.49%	y = 4.62 + 0.67x	0.99	3.82	
**SE-II**	*F*. *oxysporum*	21.72%	61.81%	66.67%	y = 4.48 + 0.97x	0.99	1.91	2.95 ± 0.97 (**P = 0.04**)
	*B*. *dothidea*	23.58%	52.99%	54.19%	y = 4.67 + 0.71x	1.00	3.13	
	*M*. *fructicola*	55.36%	70.91%	77.36%	y = 5.36 + 0.46x	1.00	0.18	
**SE-III**	*F*. *oxysporum*	47.93%	65.28%	77.78%	y = 5.27 + 0.52x	0.95	0.31	0.34 ± 0.18 (**P = 0.08**)
	*B*. *dothidea*	37.26%	65.44%	100%	y = 5.92 + 3.27x	0.78	0.53	
	*M*. *fructicola*	—	—	—	y = 0.61 + 1.78x	0.66	305.02	
**SE-IV**	*F*. *oxysporum*	—	—	—	y = 3.24 + 0.10x	0.98	—	—
	*B*. *dothidea*	—	—	—	—	—	—	
	*M*. *fructicola*	—	—	—	y = 3.26 + 0.24x	0.73	—	
**SE-V**	*F*. *oxysporum*	—	—	—	—	—	—	—
	*B*. *dothidea*	—	—	—	—	—	—	
	*M*. *fructicola*	—	—	—	y = 1.48 + 2.78x	1	19.75	
**SE-VI**	*F*. *oxysporum*	—	—	—	—	—	—	—
	*B*. *dothidea*	—	—	—	—	—	—	
	*M*. *fructicola*	—	—	—	—	—	—	
**SE-VII**	*F*. *oxysporum*	—	—	—	—	—	—	—
	*B*. *dothidea*	—	—	—	—	—	—	

**Fig 1:**
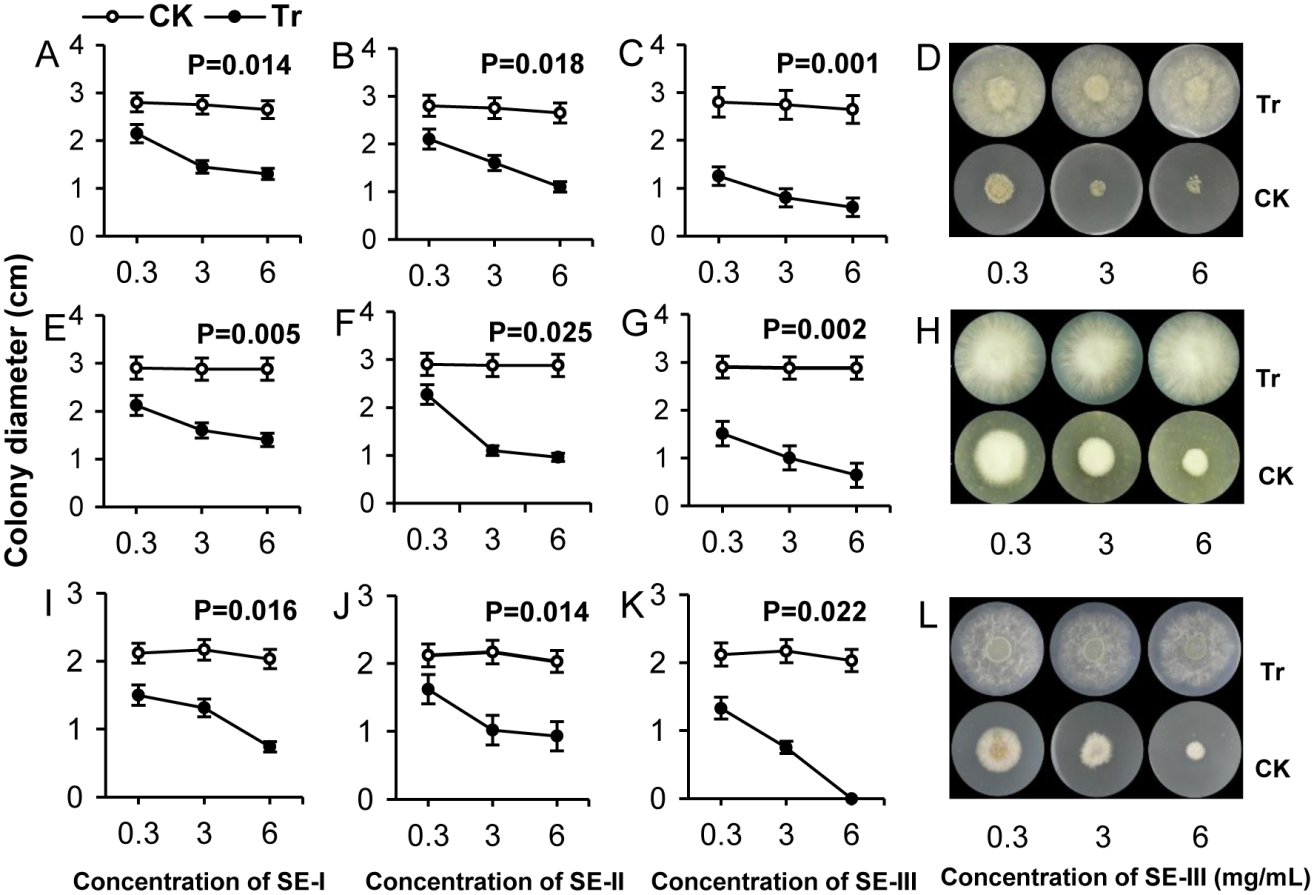
Inhibiting effects of *S*. *chinensis* extracts SE-I, SE-II, and SE-III on mycelial growth of pathogenic fungi. Inhibiting effects of *S*. *chinensis* extracts SE-I (A, E, and I), SE-II (B, F, and J), and SE-III (C, G, and K) on colony diameter of *M*. *fructicola* (A, B, C, and D), *F*. *oxysporum* (E, F, G, and H), and *B*. *dothidea* (I, J, K, and L). The data represent the mean ± SD and P values indicate a significant difference at P < 0.05. D, H, and L corresponded to C, G, and K, respectively.

For the SE-I, the EC_50_ values were 4.96, 5.54, and 3.51 mg mL^-1^, respectively, for *M*. *fructicola*, *F*. *oxysporum*, and *B*. *dothidea* (Table 2), showing that the inhibitive effect of SE-I was best on *B*. *dothidea*. For SE-III, the EC_50_ values were 0.18, 0.31, and 0.53 mg mL^-1^, respectively, and the colony diameters of the three pathogenic fungi cultured on the SE-III medium were the smallest (Fig 1: C (P = 0.001), G (P = 0.002), K (P = 0.022), D, H, L).

The inhibition rates for SE-I, SE-II, and SE-III at three different treatment concentrations (0.3, 3, and 6 mg mL^-1^) were significantly different and inhibition rate increased with larger concentration of SEs. Thus, the higher the concentration of SE-I, SE-II, and SE-III in the medium, the higher the inhibition rate impacting the growth of the pathogenic fungi. The inhibition rate of SE-III at the three treatment concentrations was higher than that of SE-I and SE-II, and reached 100% when the treatment concentration was 6 mg mL^-1^ on *B*. *dothidea* (Fig 1K and 1L).

### The effect of SE-III on the mycelial morphology of the three tested pathogenic fungi

**Fig 2:**
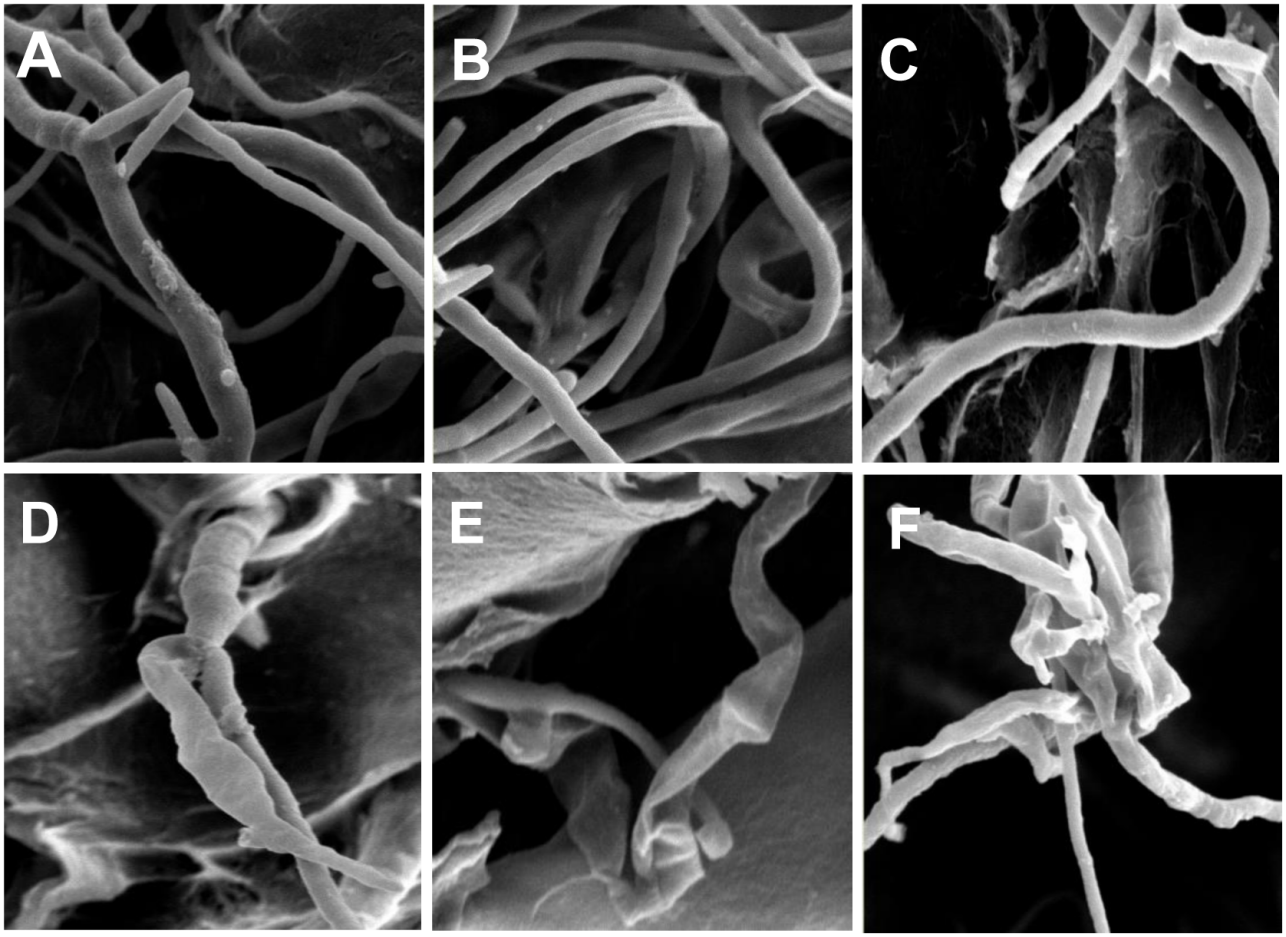
Mycelial morphology of the three tested pathogenic fungi under the electron microscope. Mycelial morphology of the three tested pathogenic fungi from SE-III-treated medium under the electron microscope. A, B, and C represent the normal condition of *M*. *fructicola*, *F*. *oxysporum*, and *B*. *dothidea*, respectively. D, E, and F represent the hypha of *M*. *fructicola* (D), *F*. *oxysporum* (E), and *B*. *dothidea* (F) from the treated medium.

When the treatment concentration of SE-III was 6 mg mL^-1^, the physiological condition of the three tested pathogenic fungi changed. Under the electron microscope (Fig 2), hyphae on the control medium were smooth and plump, having normal morphology (Fig 2A, 2B and 2C), whereas the hyphae from the SE-III-treated medium was shriveled and dehydrated, indicating that the mycelia of the pathogenic fungi presented abnormal morphological changes, such as deformation, rupture, and dissolution of the hyphae (Fig 2D, 2E and 2F). Based these results, there is direct evidence that the SEs displayed broad inhibition activity against pathogenic fungi infecting plants.

### The inhibiting effect of SEs on the spore germination and infection of pathogenic fungi

**Fig 3:**
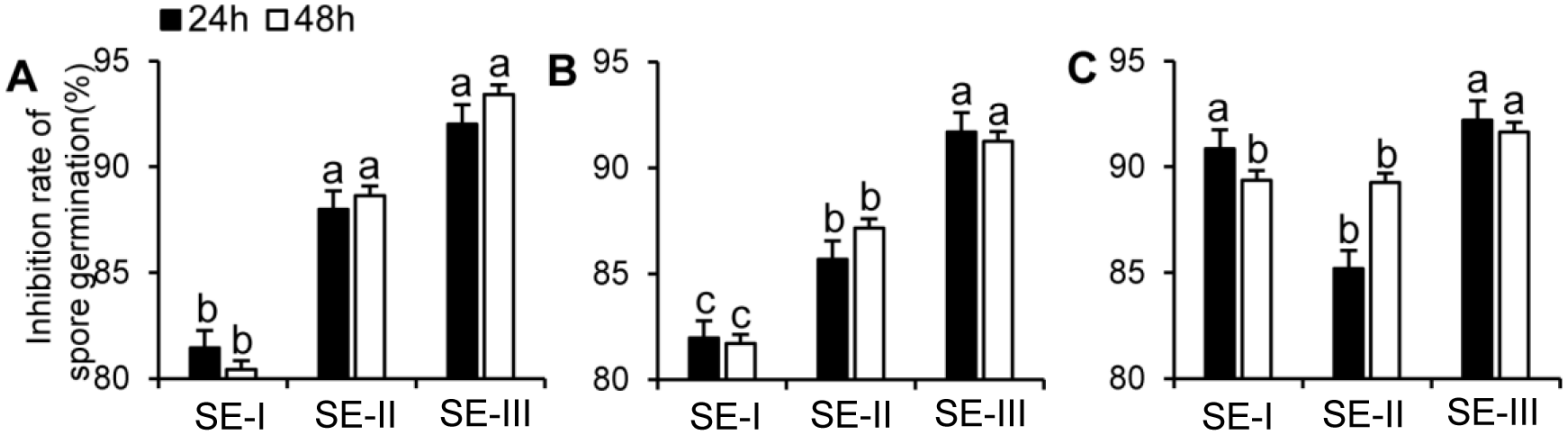
Inhibiting effect of *S*. *chinensis* extracts SE-I, SE-II, and SE-III on the spore germination of pathogenic fungi. Inhibiting effect of *S*. *chinensis* extracts SE-I, SE-II, and SE-III on the spore germination of *M*. *fructicola* (A), *F*. *oxysporum* (B) and *B*. *dothidea* (C). The black bar represents spores cultivated in the SE-containing medium for 24 h and the white for 48 h. The data represent the mean ± SD and letters indicate a significant difference (P < 0.05).

To further investigate the inhibitive effect of the SEs on the pathogenic fungi, spore germination rates of the three pathogenic fungi were calculated after the spores were treated with SEs for 24 h and 48 h. The results indicated that the SE-III treatment significantly suppressed the germination rate of the three kinds of spores after inoculation for 24 h and 48 h, and the inhibition rate reached 90% for the germination of all three kinds of spores (Fig 3C). Extract SE-I only inhibited spore germination of *B*. *dothidea* by more than 90%, whereas its inhibitive effects on spore germination of *M*. *fructicola* and *F*. *oxysporum* were not apparent (Fig 3A). The average inhibition rate of SE-II on spore germination of the three kinds of pathogenic fungi was higher than that of SE-I, but lower than that of SE-III during both treatment periods (Fig 3B). This indicated that SEs might prevent fruit from damage caused by pathogenic fungi through inhibiting spore generation of the fungi.

### The preventive effect of SEs on fruit diseases

**Fig 4:**
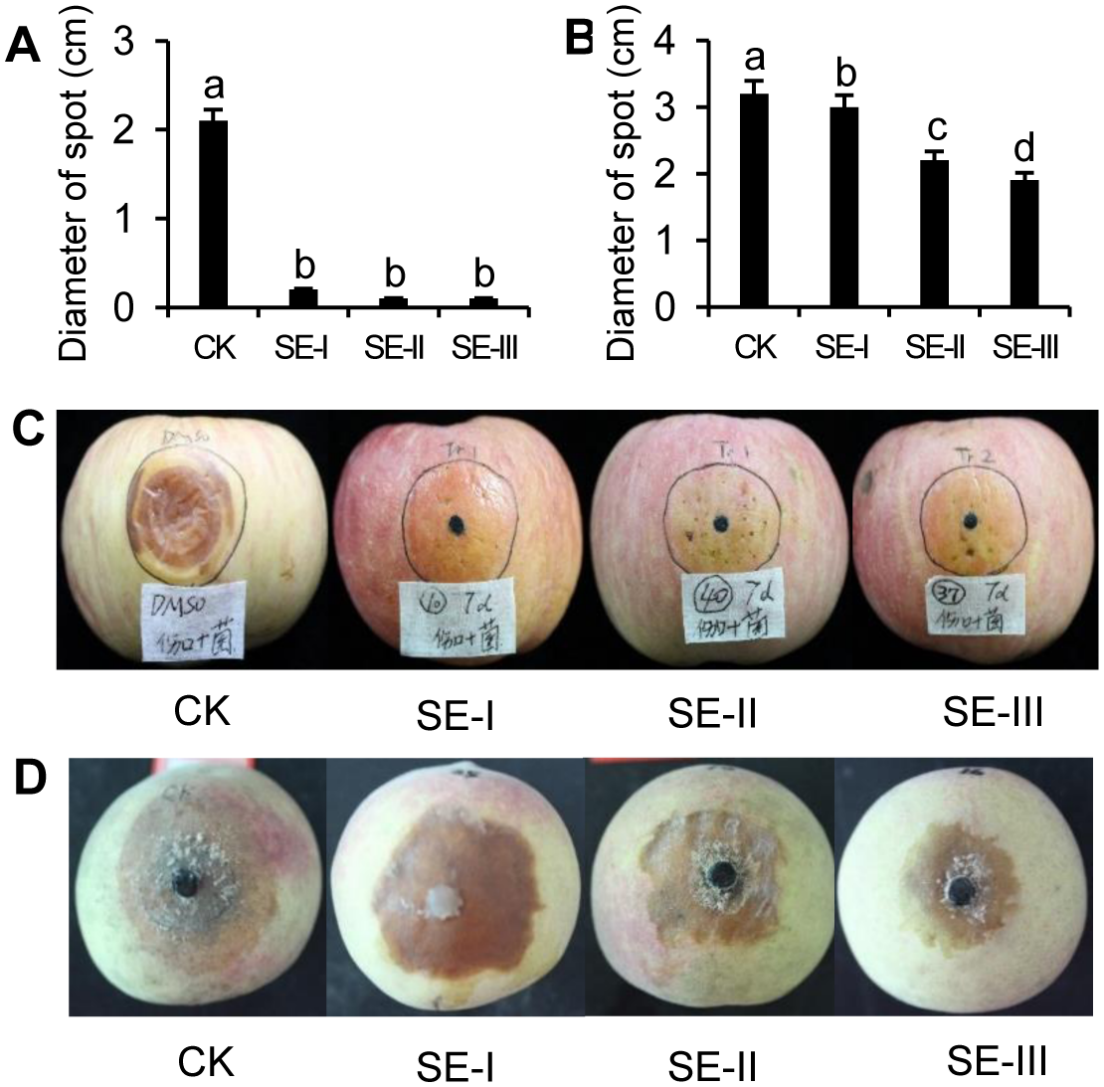
The preventative effect of *S*. *chinensis* extracts SE-I, SE-II, and SE-III on apple ring rot and peach brown rot. The preventive effect of *S*. *chinensis* extracts SE-I, SE-II, and SE-III on apple ring rot (C) and peach brown rot (D). A and B, respectively, correspond to C and D indicating the scab diameter with treatment with *S*. *chinensis* extracts. The data represent the mean ± SD and letters indicate a significant difference (P < 0.05).

The SEs were sprayed on the pericarp of apple fruits and the fruits treated with SEs tended to be more resistant to *B*. *dothidea* infection. The results indicated that the diameter of the disease spots on apples treated with SEs was smaller than those on the controls (Fig 4A and 4C). Disease spots on apples without treatment with the SEs expanded to more than 2 cm. However, the disease spots on the SE-treated apples were significantly restricted with withered fungal hyphae.

The SEs were sprayed on the pericarp of peach fruits, and the fruits treated with SEs tended to be more resistant to *M*. *fructicola* infection. Compared with the control disease spot diameter (3.2 cm), peaches treated with SEs displayed smaller disease spot diameters, which were 3 cm, 2.2 cm, and 1.9 cm for SE-I, SE-II, and SE-III, respectively, to pathogen infection (Fig 4B and 4D). Results showed that among the three treatments, SE-III performed best, with the size of disease spots on the peach fruits treated with SE-III only 50% of that on the controls. These results indicated that SEs have substantial potential for direct application to prevent fruits from infection by pathogenic fungi.

### Analysis of compounds derived from fractions of *S*. *chinensis* extract by LC-UV-ESI-MS^2^

In the study, SE-I, SE-II, and SE-III exhibited outstanding performance among the seven fractions in the preliminary toxicity experiment. Furthermore, the HPLC-ESI (±)-MS^2^ was employed to identify the compounds included in these three fractions (SE-I, SE-II, and SE-III) by standards.

From the results shown in Table 3, Schisanhenol B, Schizandrin A, Schizarin D, Schizandrin B, and isomeride Schizandrin B were found in the SE-I fraction. Schisanhenol B, Gomisin L, Schizandrin A, and Schizandrin B were in the SE-II fraction, and Schizarin D, Schizandrol A, Schizandrol B, Isoschizandrin, Schisanlignone A, Kadsulignan M, and isomeride Schizandrol B were in SE-III.

**Table 3:** Main compound types identified by LC-UV-ESI-MS^2^ in stems and leaves of *Schisandra chinensis*.

NO.	MW	[M+H]^-^^a^	MS^2^[M-H]-(m/z)^a^	Putative identifier	SE-I	SE-II	SE-III
1	386	387	[387]	Schisanhenol B	**√**	**√**	ND
2	386	387	[387]:409	Gomisin L	ND	**√**	ND
3	416	417	[417]:439	Schizandrin A	**√**	**√**	ND
4	484	485	[387]:507, 467, 436	Schizarin D	**√**	ND	**√**
5	400	401	[401]:439, 386	Schizandrin B	**√**	ND	ND
6	400	401	[401]:423, 386, 371	Schizandrin B	**√**	**√**	ND
7	432	433	[433]:415, 400, 384	Schizandrol A	ND	ND	**√**
8	416	417	[417]:399, 369	Schizandrol B	ND	ND	**√**
9	432	433	[433]:455, 415	Isoschizandrin	ND	ND	**√**
10	430	431	[431]:399, 387	Schisanlignone A	ND	ND	**√**
11	430	431	[431]:453, 387, 413	Kadsulignan M	ND	ND	**√**
12	416	417	[417]:369, 399, 384	Schizandrol B	ND	ND	**√**

ND, none detected.

√, detected.

^a,^ obtained by ion trap mass spectrometry.

### Differences in antioxidant content and profile of the related genes expression between SEs treated fruits and control fruits

**Fig 5:**
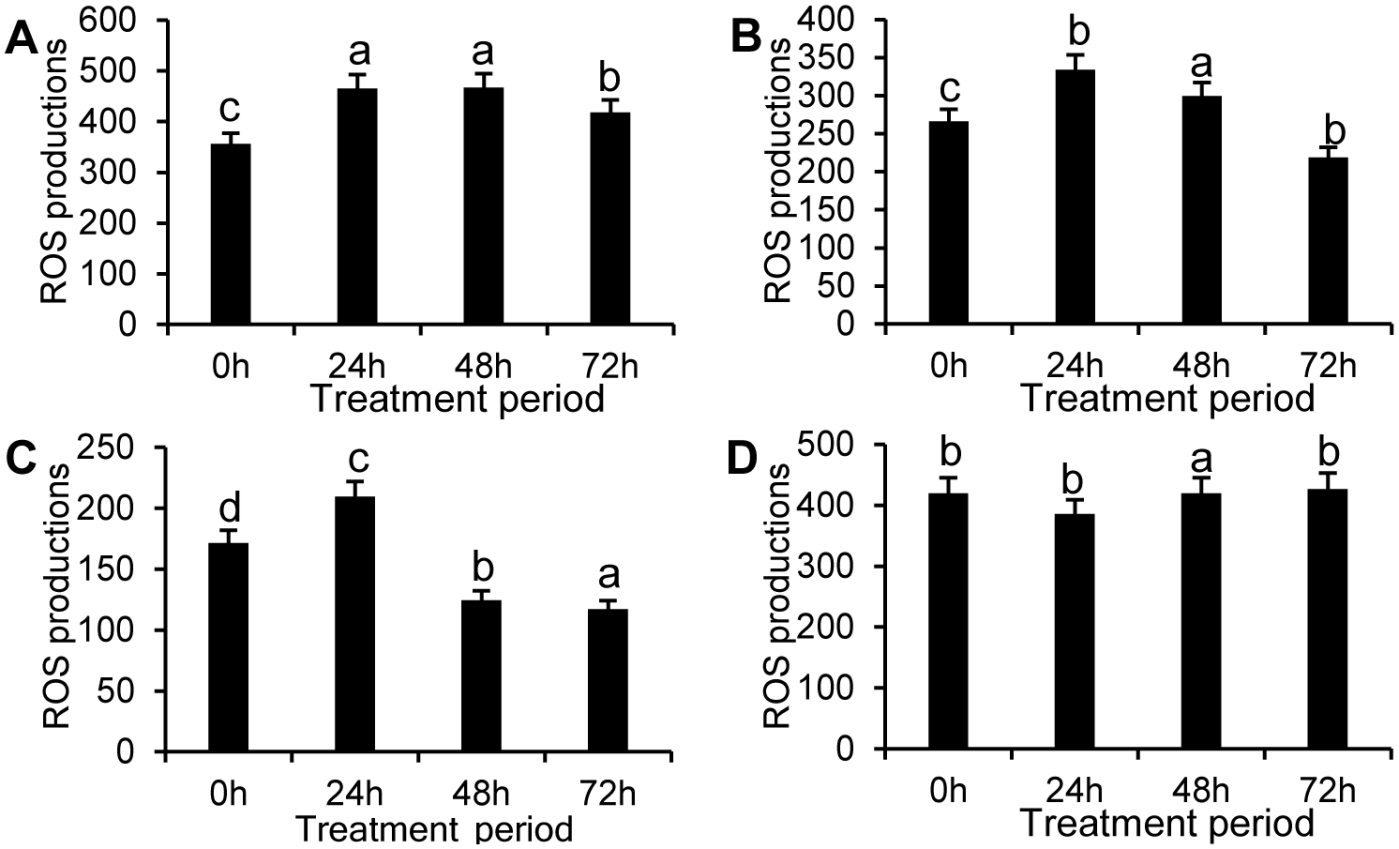
Measurement of ROS production in apple pericarps treated by the SEs. Measurement of ROS production in apple pericarps treated by SE-I(A), SE-II (B), SE-III(C), and control (D) for four different treatment periods. The data represent the mean ± SE and letters indicate a significant difference (P < 0.05).

The results showed that in the apples treated with SEs, ROS production rose in the first stage and then decreased in both the control and treatment groups, except for SE-III. However, production in treated pericarps was significantly lower than that in the control, especially for SE-III. In the SE-III treated group, ROS production reached a minimum level at 72 h. ROS production in the control group was 3.65 times that of the SE-III treated group (Fig 5).

**Fig 6:**
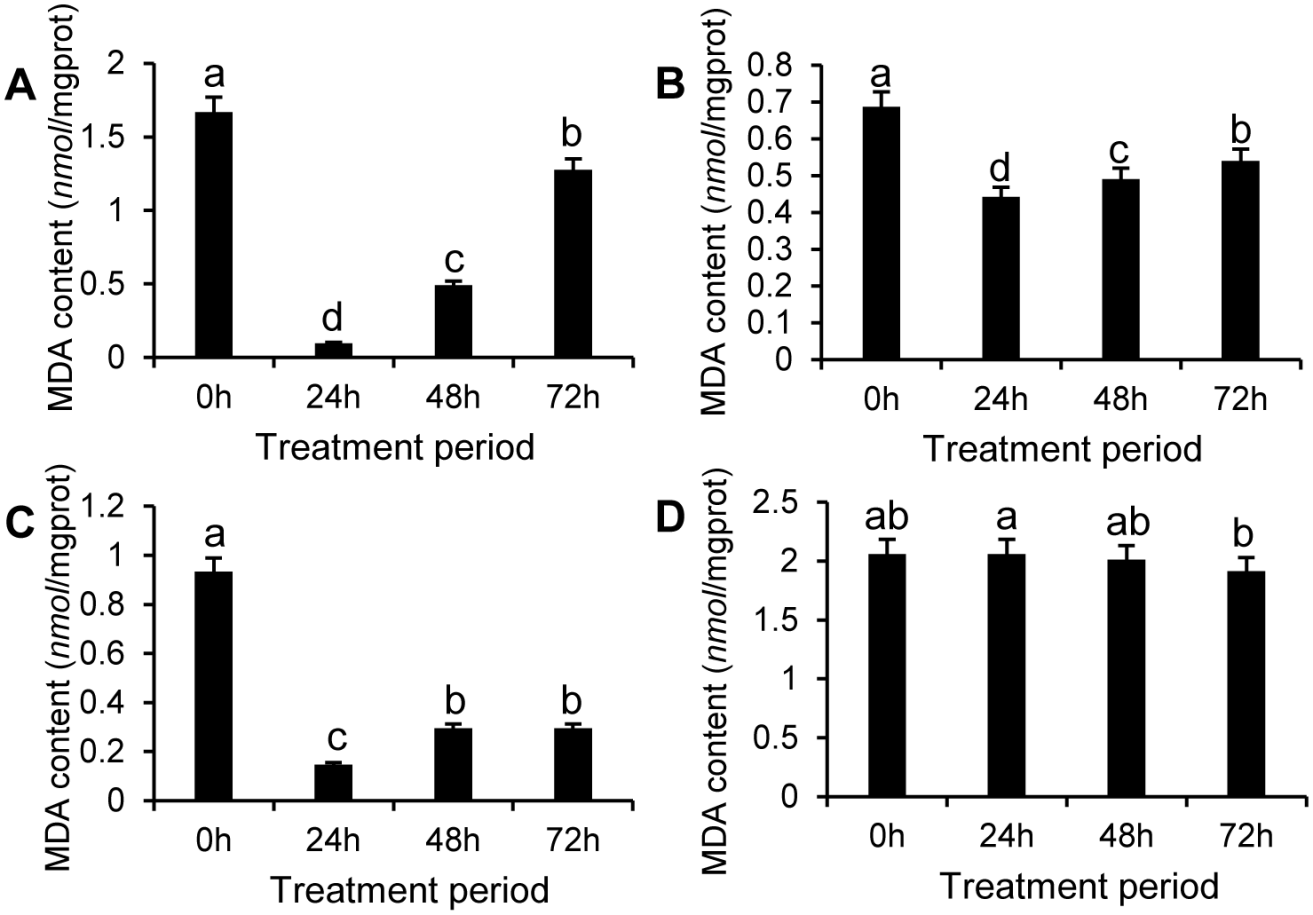
Measurement of MDA content in apple pericarps treated by SEs. Measurement of MDA content in apple pericarps treated by SE-I (A), SE-II (B), SE-III (C), and control (D) for four different treatment periods. The data represent the mean ± SE.

MDA (a product of lipid peroxidation in organisms) content in the apple pericarp first decreased, and then increased in the SE-treated groups during the treatment period, although the content was significantly lower in the treated apple pericarp at all test stages compared with that of the control (Fig 6). MDA content was lowest at 24 h in the SE-III treated group and was 6.7% percent of that of the control (Fig 6).

**Fig 7:**
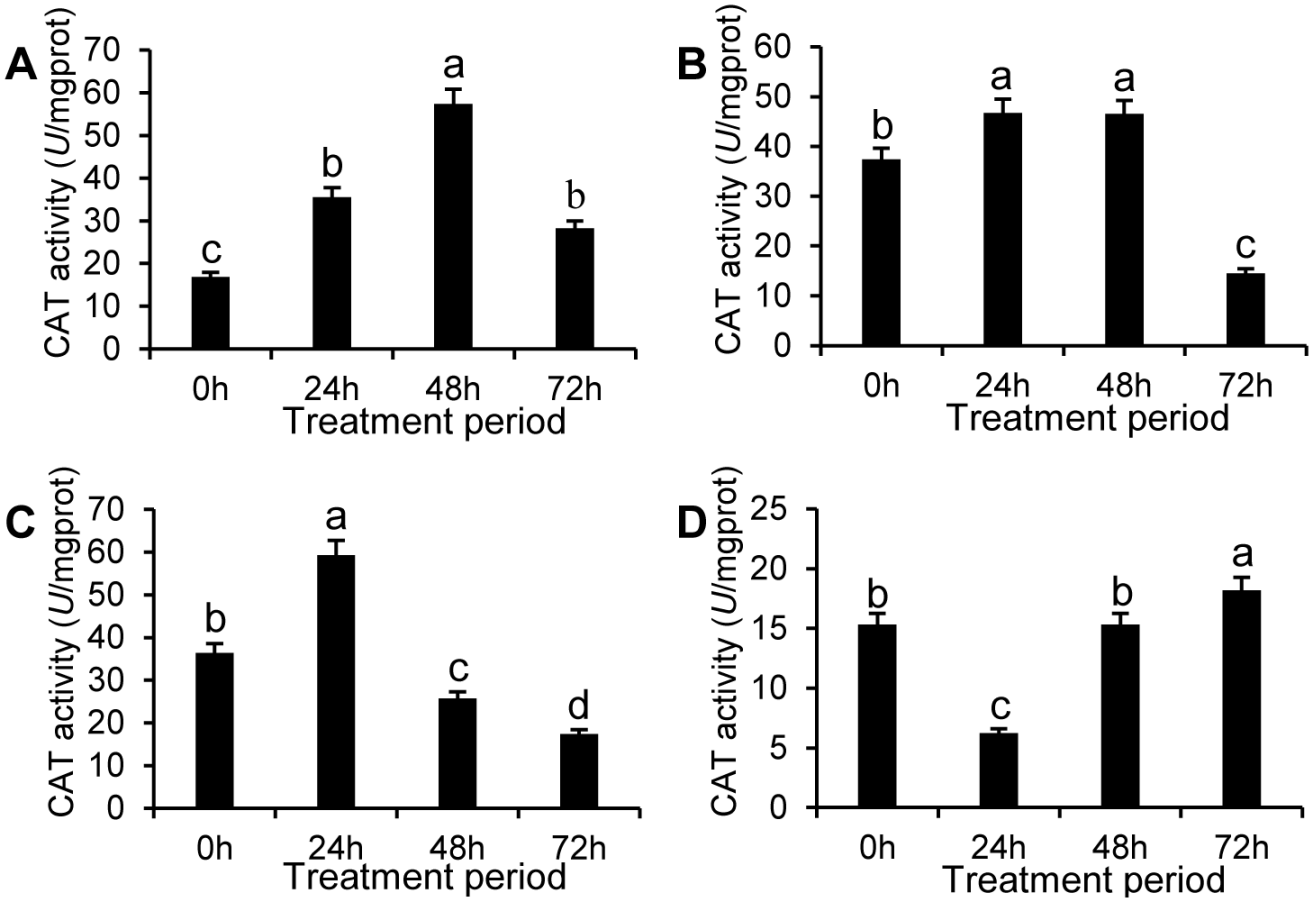
CAT activity in apple pericarps treated by SEs. CAT activity in the apple pericarp treated by SE-I (A), SE-II(B), SE-III(C), and control (D) for four different treatment periods. The data represent the mean ± SD and letters indicate a significant difference (P < 0.05).

CAT activity was markedly upgraded by the SEs and initially exhibited an increase followed by a decreasing tendency with processing time for all the treated groups. CAT activity in pericarps treated with SE-III was 2.37, 9.51, and 1.68 times that of the control during the first three sampling times, respectively, whereas there was no apparent difference between treatment and control groups at 72 h (Fig 7).

**Fig 8:**
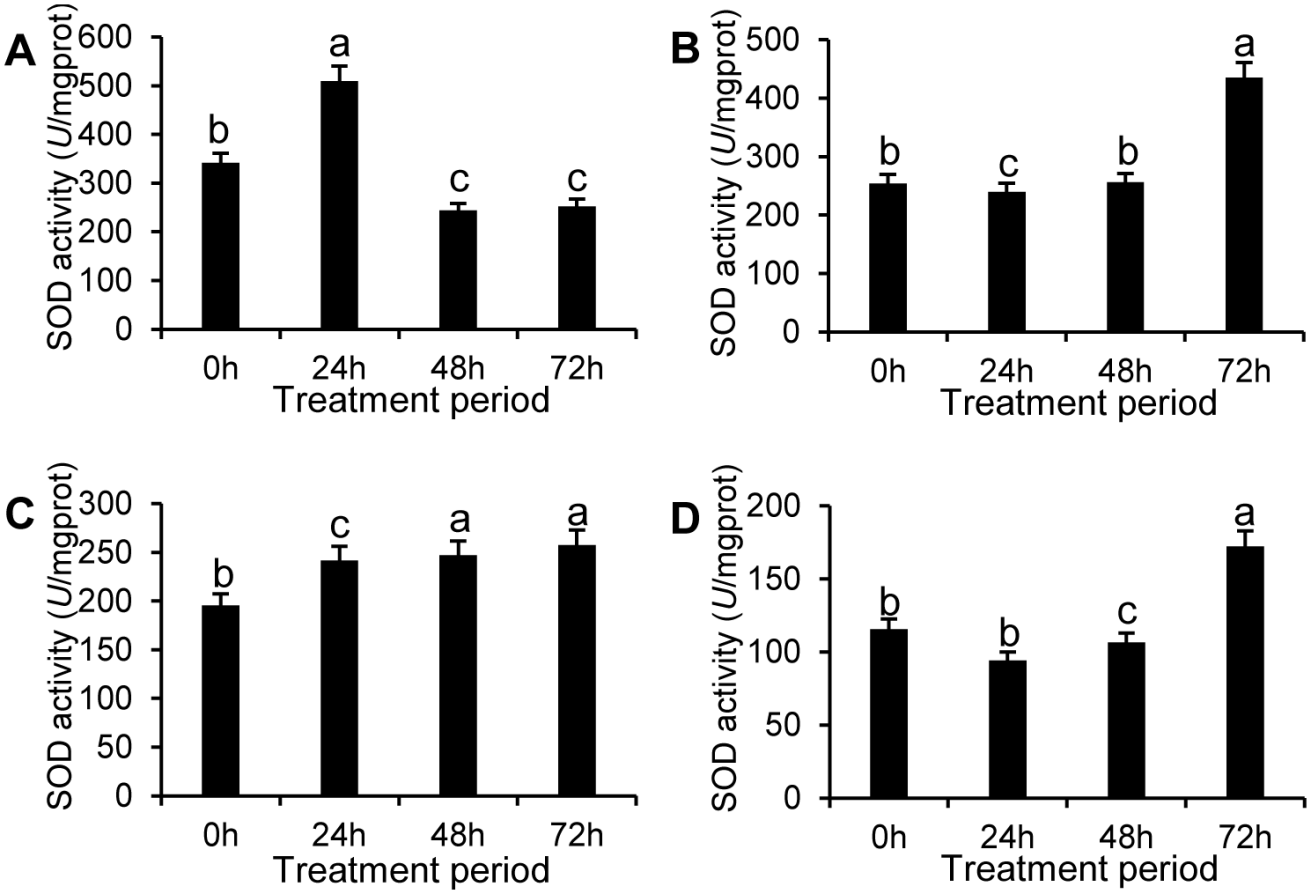
SOD activity in apple pericarps treated by SEs. SOD activity in the apple pericarps treated by SE-I (A), SE-II(B), and SE-III(C), and control group (D) for four different treatment periods. The data represent the mean ± SD and letters indicate a significant difference (P < 0.05).

SOD activity of apple pericarps treated with SEs was improved and positively correlated with time except for SE-I. In the SE-I treated group, SOD activity reached a peak and was 2.13 times that of the control group at 24 h (Fig 8). In the SE-II and SE-III treated groups, SOD activity peaked at 72 h and was 152% and 49% higher than that of the control, respectively.

The results indicated that peroxidation caused by ROS in the infected tissues rose gradually and led to MDA accumulation caused by lipid peroxidation. Furthermore, as the pathogenic fungi infected the fruit, SOD and CAT activity in treated apples rose and then, changing trend, dropped, with the activity of the two enzymes in the treated fruit displaying high performance levels compared to those of control fruits at all test stages. This indicated that SE-III possibly induced plant tissues to promote activities of preventative enzymes, inhibiting the accumulation of ROS, and then downgraded lipid peroxidation in apple pericarp tissue.

In this study, the genes related to pathogenic fungi infection and plant infection prevention were selected, and we investigated their profile in SE-III treated apple fruits. Relative gene expressions for both treated and control groups were determined by qRT-PCR, in an attempt to identify differential expression. Four genes were found to be differentially expressed between the parents, including *MdPR4*(A), *MdAFS* (B), *MdPAL* (C), and *MdPPO* (D). All were more highly expressed in the SEs treated group than in the control group and expression was also positively correlated with treatment period.

**Fig 9:**
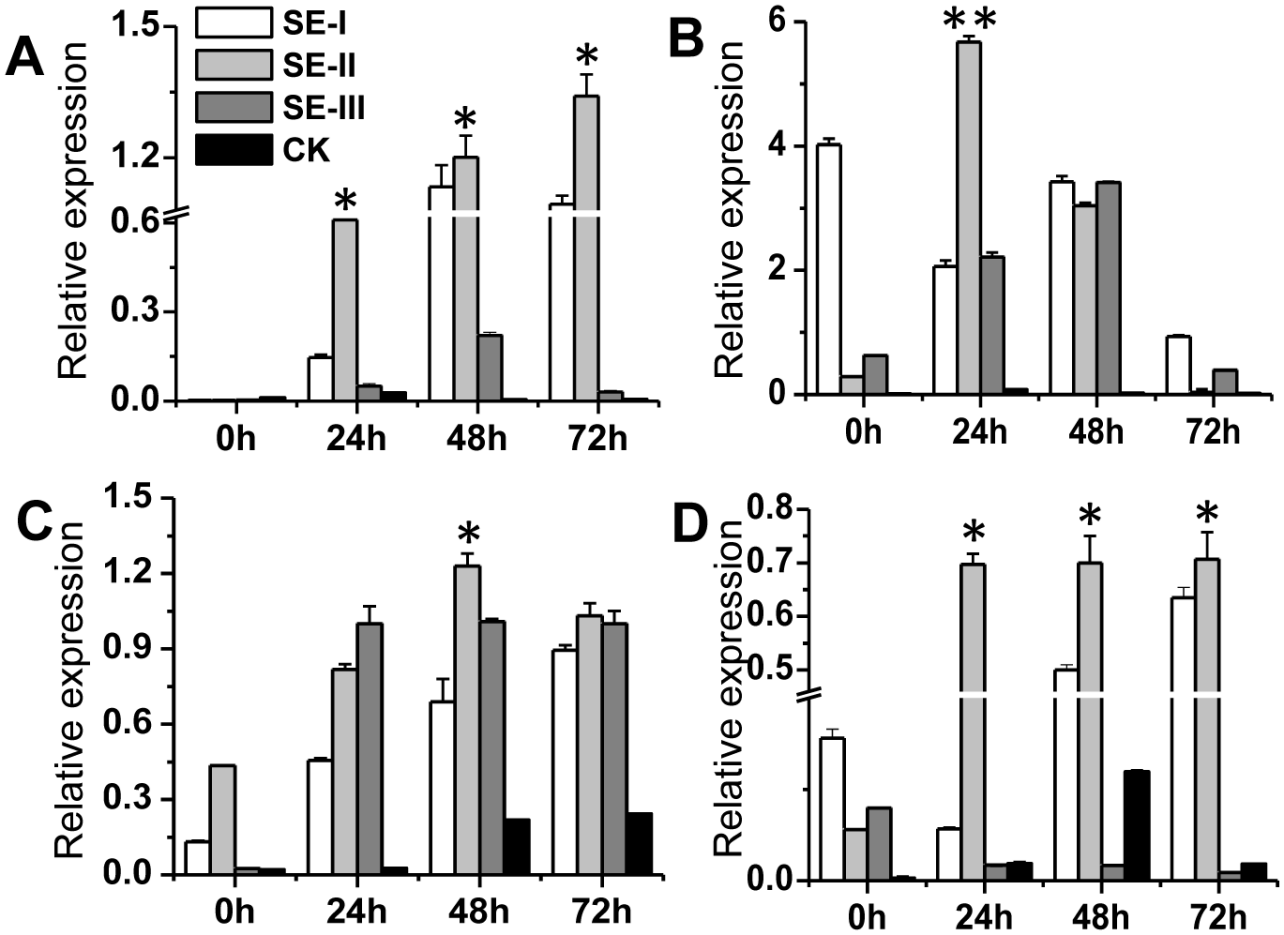
The relative expression of the four genes under treatment of three SEs. The relative expression of the four genes (A: *MdPR4*; B: *MdAFS*; C: *MdPAL*; D: *MdPPO*) under the treatment of three SEs for 4 different treatment period (0 h, 24 h, 48 h, and 72 h). The data represent the mean ± SD and asterisks indicate a significant difference (P < 0.05).

Compared with that in control groups, *MdPR4* expression in apples was significantly up-regulated by SE-I, SE-II, and SE-III during the treatment period and nearly all expression increased with time, especially for SE-II. In the SE-II treated group, *MdPR4* expression was 30.5, 5.45 and 44.67 times that of the control group at 24 h, 48 h, and 72 h, respectively (Fig 9A), although *MdPR4* expression decreased when treated by SE-III after 48 h.

Compared with that of control groups, expression of *MdAFS* was evidently up-regulated by the SEs. For example, in the SE-I treated group, *MdAFS* expression was 402, 29.43, 171, and 93 times of the control, respectively with time (Fig 9B). In SE-II and SE-III treated group, *MdAFS* expression showed a downward trend, after an initial first rise, with processing time. *MdPAL* expression increased with processing time in the SE-I, SE-II, and SE-III treated groups(Fig 9C). *MdPPO* expression was evidently up-regulated by SE-I and SE-II, whereas expression in the SE-I treated group initially increased before decreasing slightly with time. The expression of *MdPPO* in the SE-II treated group increased with processing time, and SE-III did not play a key role (Fig 9D).

## Discussion

The clinical effects of *S*. *chinensis* on pathogenic bacteria, fungi, and viruses has been widely demonstrated. However, active substances extracted from the stems and leaves of *S*. *chinensis* have been rarely reported. Whether the active substances have the potential to effectively inhibit the growth of pathogenic fungi and the potential to be a new broad-spectrum fungicide has also not yet been determined. Fruits of *S*. *chinensis* are typically chosen as experimental materials [39–42]. However, Yu (1998) reported there was no significant difference in the lignin content of the fruits, stems, and leaves [43]. Therefore, the relatively low cost and rarely studied stems and leaves of *S*. *chinensis* were chosen as our experimental material.

This study proposed a new and modern method to make full use of the previously worthless stems and leaves of *S*. *chinensis*. They not only greatly reduced extraction costs and saved resources, but also provided a new method for the future development of botanical fungicides. To obtain active substances with different polarities, different ratios of the polar organic solvent were applied to separate the primary crude extract. According to the antifungal experiment, SE-I, SE-II, and SE-III were effective, although SE-IV to SE-VII had almost no antifungal activity.

When investigating the inhibitory effects of SE-I, SE-II, and SE-III on the mycelial growth of *M*. *fructicola*, *F*. *oxysporum* and *B*. *dothidea*, the SEs show inhibitive effects against the fungi, and the effect of SE-III was the highest according to the EC_50_ values. At the same time, the inhibition rate is positively correlated to the concentration of SE applications. The inhibition rate of SE-III reached 100% against *B*. *dothidea* growth. These results were also verified by the fact that SE-III remarkably changed the growth status of test fungal hypha cultivated on the medium, causing the hypha to shrivel and dry up.

To varying degrees, the SE fractions largely suppressed the germination rates of the three kinds of spores after inoculation for 24 h and 48 h. Consistent with previous results, SE-III had the greatest effect in the experiments on spore germination. As the development and use of biological fungicide, *S*. *chinensis* extract should be further applied to postharvest disease control on fruit. Thus, this work further explored the preventive effect of SEs against pathogen invasion. As we expected, SEs can indeed prevent the invasion of pathogenic fungi to a certain degree. After spraying the SEs on the pericarp of apples and peaches, the fungal scab area of apple ring rot and peach brown rot visibly decreased. All of these factors provided evidence that the SEs can inhibit pathogenic fungi in both the *in vitro* and *in vivo* experiments. However, there was a slight injury on the surface of peach fruit. Application for pathogen defense needs to be further improved. After comprehensive analysis of its effect in fungistasis and disease prevention, our results indicated that the optimal active substance was obtained by chloroform and acetone at the ratio of 9:1.

Differences in the principle composition of the substances could be the key reason for their different efficacy. In the present study, to determine the differences in active ingredient composition among SE-I, SE-II, and SE-III, analyses of the lignans were conducted with an evaluation of HPLC-MS. According to our results, there were significant differences in lignan composition among the extracts and SE-I, SE-II, and SE-III were found to contain 5, 4, and 7 kinds of lignans, respectively. The SE-III fraction had the highest lignan abundance, and this may explain its stronger biological activity. In recent experiments on the preventative effects of lignans on fungal infection, results indicated that lignans from *S*. *chinensis* exhibited obvious inhibition and regulation of fungal infection. For example, some lignans, such as gomisin A, gomisin G, Schizandrin, and schisanhenol were recently reported to possess valuable anti-tumor promoting bioactivities by inhibiting Epstein-Barr virus early antigen (EBV-EA) activation[44], reversing P-glycoprotein-mediated multidrug resistance (Pgp-MDR) in cancer cells [45], and enhancing doxorubicin-induced apoptosis in human hepatic cancer cells, as well as through the inhibition of platelet aggregation and anti-HIV (human immunodeficiency virus) effects [46, 47]. In our study, a variety of lignans were also detected; therefore, we speculated that lignans may play a key role in suppressing pathogens. In the next experiment, we further explored physiological and molecular antifungal mechanisms.

Previous studies indicated that lignan compounds can effectively improve an organism's antioxidant levels. Research concerning SB (Schisandrin B), a major lignan isolated from S. chinensis, showed it has a high antioxidant potential in both in vitro and in vivo experiments [48–51]. Schisandrin B has been shown to prevent ischemia-reperfusion injuries in isolated perfused rat hearts by enhancing the glutathione antioxidant response and mitochondrial ATP generation capacity, or by increasing the expression levels of cardiac protective proteins, such as heat shock proteins 25 (HSP25) and HSP70. Moreover, recent studies have shown that gomisin N has anti-apoptotic activity in H9c2 cells through suppressing mitochondrial permeability transition [52, 53]. In a study on rats, You (2006) showed that SCE (S. chinensis extract) attenuates doxorubicin-induced cardiomyopathy by elevating myocardial glutathione peroxidase and superoxide dismutase (SOD) activities and reducing lipid peroxidation [54]. Extracted solutions of S. chinensis can significantly scavenge free radicals and suppress the growth of different kinds of pathogenic bacteria, such as Staphylococcus aureus, Pneumobacillus, and Enteritis bavoil [55]. In addition, SCE suppressed doxorubicin-induced toxicity by reducing ROS production and lipid peroxidation and also regulated the expression levels of glutathione metabolism and detoxification-related genes [56]. Our results were consistent with those detailed above. The study revealed that the antioxidant system of fruits was pre-induced and activated by active substances of Schisandra. It may be the most likely mechanism for postharvest fruit to effectively defend against disease pathogen invasion. Experimental results suggested that with pathogenic fungi (B. dothidea) infection, ROS production was lower in the control, especially in the SE-III treated group. MDA content was significantly lower in the SE-treated apple pericarp when compared with that of the control. SOD and CAT were widely present in the plant cells. Within a cell, SOD constitutes the first line of defense against ROS. The typical catalase reaction is the dismutation of two molecules of H2O2 to water and O2 [57]. SOD and CAT enzymes directly remove ROS to regulate its level and convert it into less cytotoxic substances. SODs are seen as a sensor of intracellular ROS [58–60]. Furthermore, SOD and CAT activity in induced apple pericarp presented higher levels compared to those recorded in the control. This indicated that the SEs might induce plant tissues to promote activities of preventative enzymes, inhibiting the accumulation of ROS and then downgrading lipid peroxidation in the infected apple pericarp.

The study found that tested genes (*MdPR4, MdAFS, MdPPO, and MdPAL*) related to the defense reaction were significantly up-regulated by the SEs. Previous studies have shown that the pathogenesis-related protein gene PR, as the terminal gene in the plant defense pathway, produces a variety of ROS and toxic secondary metabolites that cooperatively kill pathogens, and some PR gene over expression can significantly improve plant disease resistance [61]. There are other functions of PR genes, such as those involved in senescence [62], osmotic stress and mechanical injury [63]. *MdPPO* (a polyphenol oxidase gene) is considered to be the plant defense protein [64–67] and plays an important role in plant defense responses. *MdPAL* (phenylalanine ammonia lyase) plays a catalytic role in flavonoid biosynthesis, and flavonoids have antibacterial, antiviral, anti-allergic, anti-inflammatory, vasodilatation inhibiting, and other biological functions[68]. Moreover, flavonoids can also inhibit lipid peroxidation and the enzyme systems [69], as α-farnesene synthase (AFS) is the ultimate limiting enzyme in the synthesis of α-farnesene plant mevalonate pathway [70].

The present study indicated that spraying with SEs up-regulated the expression of the four genes to different degrees during the treatment period. We speculated that the active ingredients in the fractions from *S*. *chinensis* plants not only induced the up-regulation of the four genes, but also activated the antioxidant system in fruits to prevent infection, although the response of the plant itself occurs in case of infection.

Fruit quality characteristics in the process of maturity directly affect the economic value of the commercial crop. However, during this period several physiological disorders may occur. Fruit need to be induced to be resistant to infection through promoting preventative enzyme activities and regulating other scavengers or protectors, decreasing ROS production and reducing lipid peroxidation. This mechanism may extend storage time of fresh apples. According to this study, fractions from *S*. *chinensis* plants could improve antioxidant levels and promote the capacity for self-regulation while directly inhibiting disease and infection. This indicates that compounds of *S*. *chinensis* can be used as preservative or protective agents for future commercial development.

Herbal ingredients and their chemically modified derivatives have been invaluable resources for the development of therapeutic substances [71]. Technological advances in lead-generation strategies have drawn notable attention to the chemical complexity and diversity of herbal ingredients [71]. Accordingly, bioactive lignans may be a promising source of new-lead compounds for future drug development. On the other hand, lignans could be exploited as chemical tools for the starting point of new scientific discoveries—assisting in the dissection of complex phenomena into their constituent parts and the identification of the hidden molecular targets and mechanisms underlying pathophysiological phenotypes. Extracts of *S*. *chinensis* as promising resources for the development of safe, effective and multi-targeted agents against pathogenic fungi, will offer us a novel insight into future challenges and perspectives on *S*. *chinensis* research and future clinical investigations and strategies.

## Conclusions

The results described in this work showed that active ingredients were extracted with 70% acetone in *S*. *chinensis* and were separated with different organic solvents. The fraction SE-III-separated by a ratio of chloroform to acetone of 9: 1 was the most potent and antifungal active substance extracted. *S*. *chinensis* extraction was verified to successfully suppress the growth of three tested pathogens, inhibit the germination of fungal spores, and protect apple fruit from infection by pathogenic fungi. A possible mechanism to explain the results may be that SEs induced and activated the antioxidant system and also up-regulated defense-related genes. Thus, active ingredients in plants could play a major role in the development and exploitation of botanical fungicides.

## Supporting Information

S1 Table Genetic sequences of primers involved in this study. (XLSX)

## Abbreviations

SE: *Schisandra chinensis* Extract
CAT: Catalase
SOD: Superoxide dismutase
MDA: Malondialdehyde
ROS: Reactive oxygen species
PAL: Phenylalanine ammonia lyase
PPO: Polyphenol oxidase
AFS: α-Farnesene synthase
